# Supplementary material for: The genome sequence of a muscid fly, Hydrotaea militaris (Meigen, 1826) (Diptera: Muscidae)
Source: Wellcome Open Res. 2026 Aug 27;11:549. [Version 1] doi: 10.12688/wellcomeopenres.27391.1 (PMC13562971; doi:10.12688/wellcomeopenres.27391.1)
Supplement: Peer review history

# Reviewer report: Reviewer response for version 1

**DOI:** 10.21956/wellcomeopenres.30146.r166268

**Published:** 2026-09-04

**Authors:** Hans-Peter Fuehrer

**Affiliations:** 1 University of Veterinary Medicine, Veinna, Austria

The authors analyzed the genome sequence of a muscid fly, *Hydrotaea militaris* (Meigen, 1826) (Diptera: Muscidae). Overall the quality of the manuscript is fine.

 Is this species only present in Britain and not in the rest of GB / Ireland?

 Please provide Information how the specimens were sampled?

Are sufficient details of methods and materials provided to allow replication by others?

Yes

Is the rationale for creating the dataset(s) clearly described?

Yes

Are the datasets clearly presented in a useable and accessible format?

Yes

Are the protocols appropriate and is the work technically sound?

Yes

Reviewer Expertise:

Medical Entomology

I confirm that I have read this submission and believe that I have an appropriate level of expertise to confirm that it is of an acceptable scientific standard.

# Reviewer report: Reviewer response for version 1

**DOI:** 10.21956/wellcomeopenres.30146.r166269

**Published:** 2026-09-03

**Authors:** Laurence Despres

**Affiliations:** 1 Université Grenoble Alpes, Auvergne-Rhône-Alpes, Grenoble, France

The authors report on the chromosome-level genome assembly of a female specimen of a muscid fly, *Hydrotaea militaris* (Diptera; Muscidae). The assembly contains two haplotypes with total lengths of 1 703.03 megabases and 1 760.39 megabases. Most of haplotype 1 (96.2%) is scaffolded into 6 chromosomes. Sex chromosome(s) could not be identified as sequence data from the heterogametic sex was not available and homology is unreliable for sex chromosome identification in Diptera due to frequent sex chromosome turnover. Haplotype 2 was assembled to scaffold level. The mitochondrial genome has also been assembled, with a length of 18.83 kilobases.  

 The quality of the assembly was further assessed by the proportion of complete BUSCO genes recovered, which is very high: >98% of the expected gene set of diptera_odb10 reference set ( *n* = 3 285). The genome assembly is of high quality and was generated using robust and appropriate methodologies, combining PacBio HiFi long reads (32× coverage) with Hi-C data to validate and improve chromosome scaffolding. The assembly and presentation of the results follow the high standards established by the Darwin Tree of Life Project. The BlobToolKit snail plot provides a comprehensive overview of the main assembly metrics and BUSCO gene completeness.

Are sufficient details of methods and materials provided to allow replication by others?

Yes

Is the rationale for creating the dataset(s) clearly described?

Yes

Are the datasets clearly presented in a useable and accessible format?

Yes

Are the protocols appropriate and is the work technically sound?

Yes

Reviewer Expertise:

evolutionary biology, population genomics

I confirm that I have read this submission and believe that I have an appropriate level of expertise to confirm that it is of an acceptable scientific standard.

# Reviewer report: Reviewer response for version 1

**DOI:** 10.21956/wellcomeopenres.30146.r166271

**Published:** 2026-09-02

**Authors:** Rajib Deb

**Affiliations:** 1 ICAR-National Research Centre, Guwahat, Assam, India

Dataset on the genome sequence of *Hydrotaea militaris * is presented here systematically which was generated using appropriate genome sequencing techniques

 Minor concerns

 1. Please provide information on assembly curation & manual inspection for misjoins identified from Hi-C contact maps 

 2. Enlist the exact accession numbers for each raw read dataset

Are sufficient details of methods and materials provided to allow replication by others?

Yes

Is the rationale for creating the dataset(s) clearly described?

Yes

Are the datasets clearly presented in a useable and accessible format?

Yes

Are the protocols appropriate and is the work technically sound?

Yes

Reviewer Expertise:

Genomics

I confirm that I have read this submission and believe that I have an appropriate level of expertise to confirm that it is of an acceptable scientific standard, however I have significant reservations, as outlined above.

# Response

**Published:** 2026-09-03

**Authors:** Tree of Life Team Sanger

**Affiliations:** Wellcome Trust Sanger Institute, UK

Thank you for reviewing this data note.

  We think that the data note already addresses your concerns   1. Please provide information on assembly curation & manual inspection for mis joins identified from Hi-C contact maps

 In the methods, there is a section on Assembly curation, which provides information on the process for curation. It also provides a link to the Tree of Life curation resources.

   2. Enlist the exact accession numbers for each raw read dataset  

 Please note that the raw read accession numbers are given in **Table 1. Specimen and sequencing data for Hydrotaea militaris (BioProject PRJEB85208)**.  

 Tree of Life team
